# SARS-CoV-2 and its sinister routes of transmission: Mauritius from COVID safe paradise to COVID outbreak

**DOI:** 10.4314/ahs.v21i4.2

**Published:** 2021-12

**Authors:** Jared Robinson, Alexandra Leclézio, Indrajit Banerjee

**Affiliations:** Sir Seewoosagur Ramgoolam Medical College: Belle Rive, Vacoas-Phoenix, Mauritius

## Letter

SARS-CoV-2 has the capability to survive for extended periods on inanimate objects and remains infectious for up to 28 days. This invitro survival period increases transmission of the virus, as it exposes a greater cohort of the population, as it is not only transmissible by aerosols, but also by any surface.^1^

Mauritius recorded its first three cases of SARS-CoV-2 on March 18, 2020, all of which had an international travel history. On March 23, 2020 a six-week complete national lockdown of the island nation which has since been praised by the WHO commenced.^2^ By virtue of the strict and extensive nature in which the government handled the outbreak the national curfew was lifted on May 29, 2020. A total of 335 cases were detected until June 02, 2020 with a total of 10 deaths being recorded. The island nation has since been a COVID “safe” country, with no known community cases circulating within the general populous.^2^,^3^

The enhanced indomitability of the virus to survive on inanimate objects is believed to have caused the now current second wave of SARS-CoV-2 infections in Mauritius. As of March 29, 2021 the total local cases have climbed to 337. Mauritius lost its COVID “safe” status after a cluster of SARS-CoV-2 cases were detected in a fruit importation enterprise. It is believed that the initial cluster broke out in employees working at the fruit and vegetable importation facility. The virus is believed to have been imported on stock from another country, consequently infecting employees and causing an outbreak with multiple secondary clusters. One such cluster being in a school known as Curepipe College and another from a large family gathering where multiple transmissions occurred. The cases are expected to continue rising as further contact tracing takes place.^4^,^5^ The current situation developing in Mauritius should be used as a global case study, the virulence of this virus should not be underestimated as Mauritius is an example of how a nation can be shifted from COVID “safe” with no circulating active cases within the general populous to a full scale second wave of infections due to the importation and subsequent transmission of the virus from fruit packaging occurance. This should act as an international watchdog to all countries to review their importation and exportation policies and to ensure new measures are implemented in order to ensure that the virus is not imported.
